# Myelin Oligodendrocyte Glycoprotein Antibody-Associated Disease: Current Insights into the Disease Pathophysiology, Diagnosis and Management

**DOI:** 10.3390/ijms22010100

**Published:** 2020-12-24

**Authors:** Wojciech Ambrosius, Sławomir Michalak, Wojciech Kozubski, Alicja Kalinowska

**Affiliations:** 1 Department of Neurology, Poznan University of Medical Sciences, 49 Przybyszewskiego Street, 60-355 Poznan, Poland; wkozubski@ump.edu.pl; 2 Department of Neurology, Division of Neurochemistry and Neuropathology, Poznan University of Medical Sciences, 49 Przybyszewskiego Street, 60-355 Poznan, Poland; swami@ump.edu.pl (S.M.); akalinowskalyszczarz@ump.edu.pl (A.K.)

**Keywords:** neuroimmunology, myelin oligodendrocyte glycoprotein (MOG), myelin oligodendrocyte glycoprotein associated disease (MOGAD), neuromyelitis optica (NMO), NMO spectrum disorder

## Abstract

Myelin oligodendrocyte glycoprotein (MOG)-associated disease (MOGAD) is a rare, antibody-mediated inflammatory demyelinating disorder of the central nervous system (CNS) with various phenotypes starting from optic neuritis, via transverse myelitis to acute demyelinating encephalomyelitis (ADEM) and cortical encephalitis. Even though sometimes the clinical picture of this condition is similar to the presentation of neuromyelitis optica spectrum disorder (NMOSD), most experts consider MOGAD as a distinct entity with different immune system pathology. MOG is a molecule detected on the outer membrane of myelin sheaths and expressed primarily within the brain, spinal cord and also the optic nerves. Its function is not fully understood but this glycoprotein may act as a cell surface receptor or cell adhesion molecule. The specific outmost location of myelin makes it a potential target for autoimmune antibodies and cell-mediated responses in demyelinating processes. Optic neuritis seems to be the most frequent presenting phenotype in adults and ADEM in children. In adults, the disease course is multiphasic and subsequent relapses increase disability. In children ADEM usually presents as a one-time incident. Luckily, acute immunotherapy is very effective and severe disability (ambulatory and visual) is less frequent than in NMOSD. A critical element of reliable diagnosis is detection of pathogenic serum antibodies MOG with accurate, specific and sensitive methods, preferably with optimized cell-based assay (CBA). MRI imaging can also help in differentiating MOGAD from other neuro-inflammatory disorders. Reports on randomised control trials are limited, but observational open-label experience suggests a role for high-dose steroids and plasma exchange in the treatment of acute attacks, and for immunosuppressive therapies, such as steroids, oral immunosuppressants and rituximab as maintenance treatment. In this review, we present up-to-date clinical, immunological, radiographic, histopathological data concerning MOGAD and summarize the practical aspects of diagnosing and managing patients with this disease.

## 1. Introduction

Multiple sclerosis (MS) remains the most common chronic inflammatory demyelinating disease of the nervous system, so it is to no surprise that various aspects related to its pathogenesis, clinical course, outcome, treatment and epidemiology are intensively explored.

Subsequent discoveries have resulted in important clinical implications, which can be exemplified by the development of the definition of MS and related demyelinating disorders. Historical Schumacher and Poser’s criteria have been unchanged for several decades, while the current McDonald criteria have already been modified several times and do not seem definitive [1,2,3]. Moreover, for many years, patients who manifested symptoms of optic neuritis and acute transverse myelitis were treated as if this was a variant of MS–Devic’s disease, currently neuromyelitis optica (NMO). The discovery of immunoglobulin G (IgG) against the water channel aquaporin-4 (AQP-4 IgG) expressed by astrocytes, describing their role in NMO pathogenesis, distinctive clinical course, neuroimaging and neuropathological findings, all led to the recognition of NMO as an independent disease entity [4,5]. However, it quickly turned out that in 10–25% NMO patients, AQP-4 antibodies were not detected. To paraphrase Aristotle’s saying “Natura horret vacuum” science also hates “vacuum”, soon new data were published. In 21% of AQP-4 IgG seronegative patients antibodies against myelin oligodendrocyte glycoprotein (MOG) were identified [6].

In fact, anti-MOG antibodies have been extensively investigated over the last 30 years, with some in vitro, in vivo and clinical studies indicating that MOG could be an important central nervous system (CNS) antigen responsible for inducing autoimmune-mediated demyelination similarly as to in MS. It was typically found in children with acute disseminated encephalomyelitis (ADEM). The results of many further reports regarding this topic suggest that anti-MOG antibodies detection in some patients is associated with the presence of clinical syndrome different not only from MS but NMO as well. However, the role of anti-MOG antibodies in MS pathogenesis has not yet been fully excluded. It needs to be kept in mind that denaturated MOG protein could still trigger T cell immunity, even if antibodies binding MOG in its native state are absent.

In recent years criteria of the new nosological entity of MOG encephalomyelitis (MOG EN) have been postulated [7].

## 2. Myelin Oligodendrocyte Glycoprotein–Structure and Function

The myelin oligodendrocyte glycoprotein is one of several proteins produced by oligodendrocytes, which are the myelin-forming cells of the CNS. Together with others: myelin basic protein (MBP), proteolipid protein (PLP) and myelin-associated glycoprotein (MAG), MOG is an essential component of the oligodendrocyte surface membranes; these glycoproteins have fundamental roles in the formation, maintenance and disintegration of myelin sheaths [8].

MOG was first identified in the late 70s of the last century. One study has shown that another component of CNS myelin named M2, different from MBP and PLP, induced immune response which resulted in demyelination in guinea pigs [9]. In other trials, this antigen was identified as a rat cerebellar glycoprotein, which reacted with the mouse monoclonal antibodies, 8-18C5. Later it has been proven that M2 was in fact identical to MOG [10,11]. A human mature MOG is a protein containing a signal peptide of 29 amino acids followed by 218 amino acids of the mature protein [12]. This glycoprotein is expressed only in mammals with highly conserved amino acid sequence (>90%) among animal species, which suggests its important biological role. MOG belongs to the immunoglobulin superfamily, consisting of an extracellular immunoglobulin variable (IgV) domain, a transmembrane hydrophobic domain, a short cytoplasmic loop, a second hydrophobic region within the membrane bilayer, followed by a cytoplasmic end. Such structure is unique because other members of this superfamily have either a single transmembrane domain or are attached to the membrane surface by a glycolipid anchor [13].

Compared to other glycoproteins MOG is only found in relatively small amounts within myelin; however, its structure (extracellular IgV domain) and the outmost external location on myelin sheaths make it easily accessible to the potential antibodies and T-cell response involvement.

**Figure 1:**
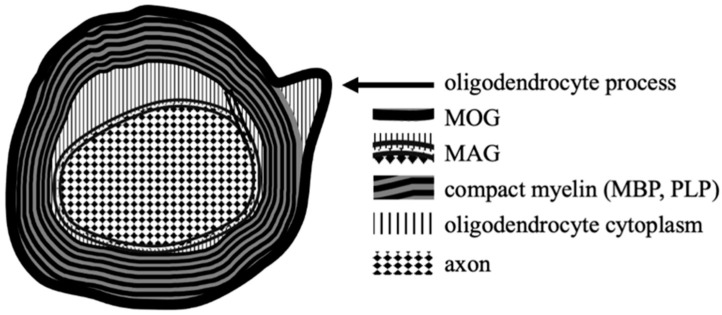
The locations of MOG and other myelin proteins on oligodendrocytes within CNS.

For other myelin constituents these interactions are much more difficult. PLP is also a transmembrane protein but has extremely hydrophobic nature and is hidden within a dense multilamellar myelin. MBP is attached to the inner surface of the cell membrane and is situated mainly in the cytoplasm. MAG is located in the innermost layer of myelin sheets, which remains in close contact with the axonal membrane [14,15,16] (Figure 1).

For some time, it has been thought that MOG locates itself on the surface of myelin sheaths and on the oligodendrocyte processes only within the CNS. However, there are anecdotal data about its low expression in the peripheral nervous system (PNS), as well [17,18]. 

MOG expression starts when myelination begins and is thus a possible differentiation marker for oligodendrocyte maturation [14]. Several essential functions of MOG are suggested: regulation of oligodendrocyte microtubule stability, maintaining the structural integrity of the myelin sheath by its adhesion features and mediation of interactions between myelin and the immune system. 

Formation of the cytoskeleton and microtubules stabilization depends on MOG interactions with MBP [19]. Experimental studies have shown that selected anti-MOG antibodies have been found to induce a significant neutral proteases-mediated loss of MBP in myelin. By contrast, antibodies to peri-axonal and structural components of myelin, such as MBP and MAG, are ineffective in inducing such MBP degradation [20]. Oligodendrocytes incubated with purified immunoglobulin G (IgG) from anti-MOG positive patients showed a striking loss of organization of the thin filaments and the microtubule cytoskeleton, which is critical for proper production of myelin [21]. The composition and function of myelin also depend on the adhesion mechanisms, which are mediated i.e., by human natural killer-1 (HNK-1) epitopes predominantly expressed on the cells of the nervous system. These molecules, identified initially as markers of adhesion and HNK cells, are associated with cell migration, neuron to glial cell interaction and outgrowth of astrocytic processes [22]. Studies revealed that a proportion of the MOG pool (together with MAG) in oligodendrocytes is glycosylated just with the human natural killer-1 (HNK-1) epitopes [23]. Therefore, MOG may play a role in the adhesion between neighboring myelinated fibers and might be a binder in the maintenance of axon bundles in the CNS. In contrast, myelin sheaths of the PNS, where MOG is rather weakly expressed, do not seem to be in contact with one another [8,10].

The localization of MOG at the external lamellae of myelin sheaths and on the surface of oligodendrocytes membrane is not the only hint suggesting this protein may be involved in the interaction with the immune system and pathophysiology of demyelinating disorders. In humans and rodents, the MOG gene is located in the major histocompatibility complex (MHC) locus. Molecules encoded by this region are found on the surfaces of cells and are involved in antigen presentation, inflammation regulation, the complement system, and the innate and adaptive immune responses. Also, the gene has a certain structural similarity to the B7-CD28 superfamily–encoded proteins are expressed on the surface of the professional antigen-presenting cells (APC) [24].

MOG can directly activate the classical pathway of the complement cascade. Reports from experimental studies suggest that binding of MOG to the C1q and C3d components can activate the complement system. The potential role of MOG in the complement cascade may give insight into the role of MOG in the demyelinating processes [25]. 

The substantial progress of MOG-dependent CNS autoimmunity research was made possible by the development of a few models of the disease–MOG EAE (MOG experimental autoimmune encephalomyelitis) in rodents. A well-defined model of autoimmune demyelination in the CNS is induced by recombinant MOG 1-125 that allowed studying the combination of pathogenic T-cell and antibody-dependent effector mechanisms. [26,27]. In other interesting animal studies, T cell receptor (TCR) transgenic mice specific for MOG spontaneously developed isolated optic neuritis without any evidence of EAE [28].

Low amounts of MOG in myelin and observed in experimental data MOG-associated demyelinating activity led to the hypothesis about the high immunogenic potential of this protein–the concept that MOG could be the highly specific autoantigen in most frequent demyelinating disease which is MS was very attractive. Especially that existent neuropathological findings in MOG antibody-positive patients were somewhat consistent–most of them revealed MS pattern II lesions with infiltrations of T cells and deposition of IgG as well as activated complement at the sites of ongoing demyelination. Thus, it supports an idea about humoral immune pathogenesis of MOG antibody disease [29].

Indeed, several clinical trials have found that antibodies against MOG would play a role in MS pathophysiology: they have been shown in active lesions in MS patients, the serum anti-MOG-Ig response has been established in both early and late stage of this disease, patients with higher anti-MOG antibodies had relapses more often and earlier than patients without or low anti-MOG levels. Moreover, it has been proven that anti-MOG antibodies were a predictor of clinically definite MS in subjects with clinically isolated syndrome (CIS) [30,31,32]. 

On the other hand, several research groups yielded contrary results. The frequency of positive samples with low titres of anti-MOG IgG was similar in MS patients and healthy subjects [33]. Another study conducted on almost half a thousand subjects proved that serum antibodies against MOG were not associated with an increased risk of progression to clinically definite MS in patients who had CIS [34]. Results of a very recent multicentre cross-sectional study involving an unselected large (n = 685) cohort of adults with MS indicated that only two (0.3%) patients were anti-MOG antibodies positive. The authors definitely stated that anti-MOG antibodies testing in typical MS presentation is valueless [35]. 

Finally, another very novel immunopathological study has proven that the deposits of activated complement components (an essential feature of MS pathology pattern II of the early active lesion), previously emphasized as a characteristic pathological finding of the anti-MOG disease, were rarely observed in perivascular areas. Most investigated lesions (153 of 167 in total) showed an ADEM-like pattern of damage: the perivenous disseminated inflammatory demyelination, MOG-dominant myelin loss with preserved oligodendrocytes, CD4-dominant T cell infiltration, and perivascular MOG-laden macrophages [36].

One of the potential explanations for equivocal findings raised by experts is the type of anti-MOG antibodies laboratory tests employed. Methods used in the initial and former studies might have insufficient specificity and sensitivity; immunoblotting and ELISA techniques do not precisely discriminate between denatured and folded proteins, also Western blotting detects antibodies directed only against continuous epitopes on denatured proteins [11,20,30,31,32,37].

**Figure 2:**
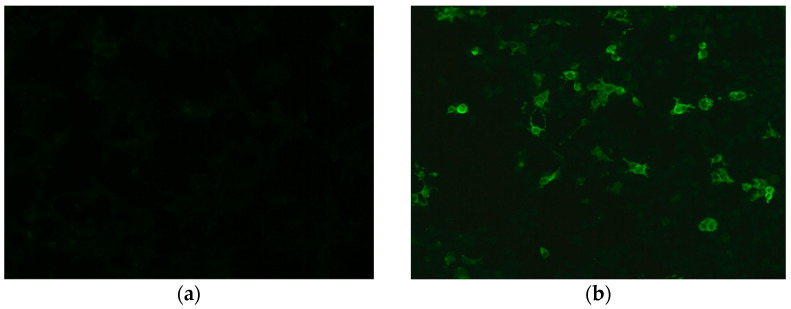
Detection of MOG IgG in a cell-based assay: (**a**) negative control stain; (**b**) positive MOG IgG reaction. Images from the collection of Division of Neurochemistry and Neuropathology, Poznan University of Medical Sciences.

Development of more specific methods ensures the reliable recognition of antibodies against native correctly folded, glycosylated MOG with preserved tertiary structure. One of them is optimized cell-based assay (CBA), a technique that maintains the conformational structure of a full-length human MOG, which allows identifying humoral immune response against this protein [38]. Another technique is radioimmunoassay in which soluble four human MOG extracellular domains are tetramerized and used for antibodies detection [39]. The tests standardization improvement is still a challenge for scientists. Very recently results of a multicenter, international study which compared the reproducibility of different antibody assays (live and fixed immunofluorescence CBA [IFT], live flow cytometry CBA [FACS] and ELISA) have been published. Live MOG-IgG CBAs showed excellent agreement for both positive and negative samples [40]. Currently, (IFT/FACS) is a golden standard test that uses whole length human MOG as an antigen and secondary antibody specific against Fc- or IgG1 to prevent cross-reactivity (Figure 2). “Double-positive” result AQP4-IgG/MOG-IgG is considered as a red flag and requires re-testing according to the 2018 International Recommendations [7].

For future research, the formulation of sophisticated analytical approach is crucial, especially that only antibodies which recognize properly folded MOG protein are pathogenic.

**Figure 3:**
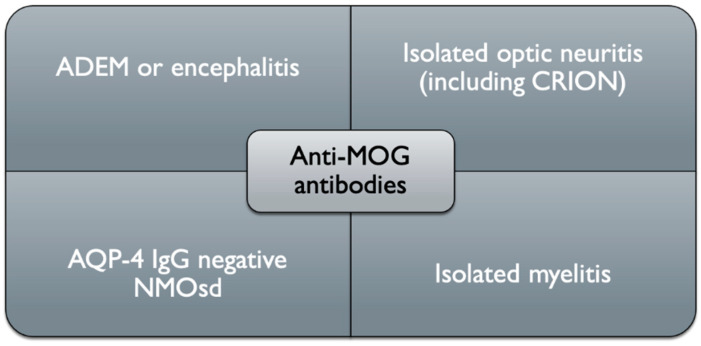
Schematic clinical representation of anti-MOG associated disorders (ADEM, acute disseminated encephalomyelitis, AQP-4, aquaporin-4, MOG, myelin oligodendrocyte glycoprotein, CRION, chronic relapsing inflammatory optic neuropathy, NMO, neuromyelitis optica).

Until now multiple research groups have detected serum anti-MOG antibodies with aforementioned optimized cell-based assays, mostly in patients with a phenotype of acute disseminated encephalomyelitis (ADEM), brainstem/cortical encephalitis, optic neuritis, (ON) (unilateral/bilateral), transverse myelitis (TM) and longitudinally extensive transverse myelitis (LETM). In other words, we can say this separate clinical entity (different terms are used: anti-MOG disease, MOG-IgG associated disorder and–most commonly nowadays–MOG antibody disease (MOGAD) is an inflammatory demyelinating condition of the CNS characterized by a mono- or multiphasic course of neurological deficits, which does not meet the criteria for typical MS or other known neuroinflammatory illnesses (especially neuromyelitis optica spectrum disorders, NMOSD) and occurs in the presence of MOG antibodies [7,41] (Figure 3). 

## 3. Clinical Picture

### 3.1. ADEM Presentation

One of the initial research reports which employed highly specific assays, identified a humoral immune response against MOG, particularly in monophasic (rarely in multiphasic) ADEM pediatric patients [39]. Other studies confirmed observations that ADEM symptoms: systemic (fever, headache, nausea, vomiting, malaise, altered mental status) and more specific, which vary based upon the locations of the lesions within the CNS (vision impairment, ataxia, hemiparesis, hemisensory loss), dominate in young age patients. Anti-MOG antibodies were present in 40–68% of children with ADEM diagnosis [42,43,44,45,46,47]. In adults with the positive anti-MOG test, ADEM presentation is less frequent, varies from a few up to 18% of cases [45,48]. Ongoing myelin development and compaction likely have an important influence on ADEM’s clinical picture of MOG antibody disease. As mentioned, the presentation of this condition in children is mostly ADEM or ADEM-like (multiphasic disseminated encephalomyelitis (MDEM), ADEM–optic neuritis) and evolves with age to “optico-spinal syndrome” (optic neuritis, myelitis and brainstem encephalitis) in adults.

### 3.2. Optic Neuritis Presentation

Optic neuritis (ON) is the most frequent clinical phenotype in older age patients. In the two largest clinical trials conducted in France and United Kingdom (UK), anti-MOG disease onset with ON involved about 44–60% of subjects [45,46,48]. In some studies as much as 88% patients had acute ON at least once [49]. Young cases frequently developed unilateral ON, while in patients with the late onset of disease both optic nerves were inflamed [46]. Most patients with MOG-IgG optic neuritis demonstrate a severe vision loss with eye pain at the onset, but favorable vision outcomes. Recurrent optic neuritis is referred to as chronic relapsing inflammatory optic neuropathy (CRION) [50]. 

### 3.3. Transverse Myelitis Presentation

Isolated transverse myelitis (TM) was the initial presentation of MOGAD in about 20% patients, but a combination of TM and ON occurred in additional 8 to 15% of subjects [45,48,51]. Quite distinctive, and similar to NMO, symptoms relate to large inflammatory lesions, longitudinally extensive transverse myelitis (LETMS), especially in young age. In this condition, injury usually involves three or more adjacent vertebral segments of the spinal cord. However, in contrast to NMO, low parts of the spinal cord are inflamed, including medullary cone, which results in acute flaccid myelitis (ATM) syndrome. Besides symptoms like tetra-/paraparesis, dysesthesia and pain, the urinary retention/incontinence and/or bowel and/or erectile dysfunction developed at least once in almost 70% patients with TM [49].

### 3.4. Other Manifestations (Brainstem and Cortical Encephalitis)

The manifestation of brainstem encephalitis in the course of the MOGAD includes features suggesting MS, such as dysarthria, dysphagia, internuclear ophthalmoplegia, third nerve palsy with diplopia, nystagmus, trigeminal hypesthesia, facial nerve paresis–in one report they affected 30% of subjects [52]. Lesions within the dorsal medulla oblongata can also cause intractable nausea, vomiting, hiccup and cough-symptoms of the area-postrema syndrome. This condition, previously thought to be highly specific for NMO, persisted in about 15% of the anti-MOG positive patients in UK study; most of them (91%) were presented at onset of attacks [45].

Cortical inflammation associated with the MOGAD manifests mostly with epileptic seizures, sometimes also with disturbances of consciousness and behavior. This encephalogenic phenotype of the anti-MOG disease seems seldom, clinical trials reported anecdotal cases of seizures [45,48]. However, studies comparing the incidence of acute epilepsy attacks in the MOGAD and NMOSD patients showed that seizures are more than 20 times frequent in the former disorder [53]. Similarly, epilepsy symptoms in MS are somewhat unusual; a comprehensive review article stated that seizures occur only in about 2–3% of all patients with this condition [54]. 

Cortical encephalomyelitis treatment with steroids is effective, so usually the prognosis is very good. Unfortunately, the fulminant demyelinating encephalitis with a fatal outcome was also reported recently [55].

In 2018 based on experts’ consensus the proposal of the MOGAD diagnosis criteria was published [7]. Disease confirmation includes three necessary components:

- clinical picture,
- neuroimaging (MRI) or neurophysiological exam (in ON optical coherence tomography or visual evoked potentials) findings indicating demyelinating injury within CNS,
- biochemical (the positive result of MOG IgG test performed with modern cell-based assay).

In the differentiation process CSF analysis could be very useful. Detailed findings such as cell number, protein concentration, and immunoglobulins in patients with MOGAD, NMOSD, and MS were summarized in Table 1.

## 4. Disease Course and Epidemiology

In general, patients suffer from the above presented isolated syndrome, but sometimes a combination occurs: TM + ON, brainstem + cortical encephalitis, brainstem encephalitis + ON, ADEM + ON.

There are several differences when comparing patients with MOGAD and NMO. MOGAD occurred more commonly in Caucasians, whereas AQP4-seropositive NMO was found predominantly in non-Caucasian populations. NMOSD had a relatively stronger female predilection with a ratio of about 8:1 while MOGAD showed a lower ratio range. MOGAD appeared to have an earlier age of onset compared to NMO. Finally, despite a frequently relapsing disease course, patients with MOGAD had an overall milder disease course compared to NMO patients [45,48,49].

Based on initial observation MOGAD was considered monophasic, which might be true in young age; most of the antibody-positive children experience only one incident of the disease [48,60]. Further studies with follow-up have shown that in adults the disease usually has multiple attacks. Relapses were most frequent in patients who presented ON symptoms and occurred in the months up to 1–2 years after the onset attack [44,45,49,53]. 

## 5. Neuropathology

Based on a study of two autopsies and 22 brain biopsies of clinically, radiologically and serologically confirmed MOGAD cases, Höftberger et al. concluded that MOGAD pathology was characterized by coexisting perivenous and confluent white matter demyelination [60]. This suggests a transitional pattern of MOG antibody associated demyelination-between MS, where demyelination is confluent, and ADEM, where it has a perivenous pattern. The inflammatory infiltration consisted mainly of CD4+ T cells and granulocytes, in contrast to MS where CD8+ T cells predominate. Compared to MS, intracortical rather than leukocortical demyelinated lesions were more common. Importantly, AQP-4 was preserved, as MOGAD is not an astrocytopathy. Complement deposition within active lesions was observed, but not on astrocytes or glia limitans. Contrary to expectations, MOG was not preferentially lost. MOGAD pathology shared features with ADEM and MS, but could be easily distinguished from AQP4-positive NMOSD and MS.

## 6. Neuroimaging

Imaging of the optic nerves is essential in the diagnosis, as ON is the most common presenting feature of MOGAD. MRI features of ON attacks, which were highly reproducible among these patients, included anterior optic pathway lesion with distinct optic nerve head swelling and injury of the retrobulbar nerve segment. Usually, the optic chiasm and the optic tract were spared. In turn, most NMO patients with ON had a posterior optic pathway and intracranial portion of the optic nerve and chiasm involved [61,62]. In another study, a symptomatic feature of the MOGAD and NMO was bilateral and longitudinally extensive ON, in contrast to MS, in which ON was mostly unilateral, with shorter segment optic nerve damage [62].

MRI of the spinal cord in TM patients revealed two potential lesion patterns. The first, mentioned earlier is LETM–characteristic extensive involvement of the spinal cord with abnormal hyperintense T2 signal within at least three contiguous vertebral body segments and involving more than 50% of the axial section of the spinal cord with its swelling. This MRI picture might develop in NMO as well, but in the course of MOGAD the pathology was more frequently confined to gray matter, presented as a sagittal line/H sign: sagittal line hyperintensity surrounded by a T2-hyperintense signal, concurrent with an H-shaped hyperintensity seen in axial sequences and due to grey matter involvement [63]. The second type of injury is a T2-hyperintense change affecting less than two vertebral segments [52,62]. Lesions can be visible along the entire spinal cord, but the localization in a medullary cone is believed to be highly specific for MOGAD diagnosis [52]. 

Brain imaging is considered abnormal in about half of the patients [48]. In ADEM patients T2-weighted and fluid-attenuated inversion recovery (FLAIR) sequences of brain MRI typically revealed an unspecific picture, marked by bilateral, poorly delineated, blurred and extensive lesions involving juxtacortical white matter, deep grey matter and rarely cortical grey matter [64]. Recently, MRI images of unilateral cortical T2-FLAIR hyperintense changes, without the involvement of the adjacent juxta-cortical white matter, were reported in patients with cortical presentation (seizures) of MOGAD. They were called FLAMES (FLAIR-hyperintense lesions in anti-MOG-associated encephalitis with seizures) sometimes associated with FLAIR-variable unilateral enhancement of the leptomeninges (FUEL) [65,66].

Other characteristic lesions for MOGAD (although still not highly specific as they can also appear in NMO) were poorly delineated (“fluffy” or “fuzzy”) areas located infratentorially: in the brainstem, cerebellar peduncles or the area postrema, sometimes adjacent to the fourth, the third ventricle and the aqueduct. Typically, the number of changes was lower than three [62].

No highly specific radiological presentation has been identified to differentiate MOGAD from non-MOG antibody cases. In the diagnostic process, the recommended “red flags” on brain MRI can be helpful to rule out MOGAD: ovoid/round lesions in the periventricular area, Dawson’s finger-type, juxtacortical U fibers, and T1 hypointense lesions are all typical features of MS [7,67].

**Figure 4:**
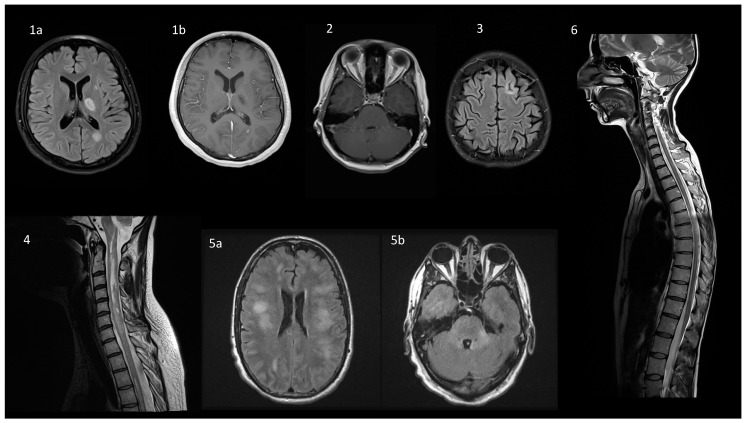
Typical magnetic resonance findings in a spectrum of diseases overlapping with MOGAD. (**1**) ADEM presentation in a 17-year old female with behavioral changes and transient right-sided paresis. On axial FLAIR (**1a**), a periventricular lesion at the level of the left subcortical nuclei (17 × 11 mm) and the left ventricular triangle (15 × 10 mm) are visualized. On axial post-contrast T1-weighted image (**1b**) slight contrast enhancement is present in the latter. (**2**) Optic neuritis in a 26-year old female with AQP4-ab positive NMOsd: bilateral enhancement of the optic nerves on axial T1-weighted post-contrast sequence. (**3**) Cortical involvement in a 22-year old female with MS: on axial FLAIR there is a 4 mm long linear hyperintensity that surrounds the cortical gyrus and involves both, the cortex and subcortical white matter. (**4**) AQP4-ab positive NMOsd in a 43-year old female: on a sagittal T2-weighted sequence LETM is visualized from C1–C2 to C5. (**5**) MOGAD in a 47-year old male presenting with ADEM with confirmed MOG-ab seropositivity. On axial FLAIR images there are diffuse nonspecific white matter hyperintensities involving both cerebral hemispheres, corpus callosum (**5a**), posterior fossa (**5b**), but periventricular lesions are lacking. (**6**) MOGAD in a 34-year old female presenting with spastic paraparesis, which improved significantly after a 5-day intravenous methylprednisolone course: on a sagittal T2-weighted sequence multiple hyperintensive poorly demarcated lesions are visible throughout the spinal cord, including the cervical and thoracic regions, and affecting medullary conus, as well. The lesions are not visible on T1-weighted sequence and are non-enhancing. Abbreviations: MOGAD—MOG-antibody associated disease, ADEM—acute disseminated encephalomyelitis, FLAIR—Fluid Attenuated Inversion Recovery, NMOsd—neuromyelitis optica spectrum disorder, AQP4—aquaporin 4, LETM—longitudinally extensive transverse myelitis. Subfigures 1a,b, 2–4 and 6: from the collection of the Department of Neurology, Poznan University of Medical Sciences, Poznan, Poland. Subfigure 5a,b: courtesy of dr Claudia F. Lucchinetti, Department of Neurology, Mayo Clinic, Rochester, MN, USA

These imaging criteria could differentiate MS from NMO and MOGAD patients, with high sensitivity and specificity. Unfortunately, the separation of both antibody-associated CNS diseases by MRI was less accurate. Therefore, researchers look for any possibility to improve this issue. Very recent quantitative analysis of neuroimaging changes has brought promising results. In comparison with the NMO group, the brain lesions of the MOGAD patients was of a larger size, dispersed distribution, and higher probabilities in the cerebellum, pons, midbrain, and gray matter and juxtacortical white matter in temporal, frontal, and parietal lobes [68]. Examples of MR images associated with different clinical presentations of MOGAD are depicted in Figure 4.

## 7. MOGAD Diagnosis

Resolving the NMO and MOGAD differentiation issue is also not facilitated by the fact that they both share a number of clinical manifestations, although they are autonomous nosological entities–NMO is an autoimmune astrocytopathy, with CNS demyelination secondary to the astrocytic destruction [5]. 

Thus, from the practical point of view, none of the clinical and neuroimaging finding is highly specific for MOGAD. The result of anti-MOG antibody testing is crucial for appropriate diagnosis, therefore the international guidelines regarding accurate analysis are detailed. The golden standard method for experts is the cell-based assay with full-length human MOG as target antigen [7]. They also suggest employing Fc or IgG1- specific secondary antibodies to avoid cross-reactivity with IgM and IgA antibodies. Detection of anti-MOG IgG antibodies by other laboratory methods, like immunohistochemistry, peptide-based ELISA or Western blotting, is not recommended due to their low specificity. The optimal source for MOG IgG testing is serum, since antibodies concentrations are low in cerebrospinal fluid. Samples should be kept and shipped at 4 °C or on dry ice if arrival is not possible within 1–2 days. 

Another vital concern is sample timing. MOG-IgG serum levels depend on disease activity, with higher median concentrations during acute attacks, and low during remission or in the chronic phase or even absent after a monophasic incident [52]. Treatment strategy also influences the test results. Therefore, when MOGAD is highly suspected but with negative anti-MOG- IgG testing, patients should be retested, optimally during acute attacks, treatment-free intervals, or 1–3 months after plasma exchange or intravenous immunoglobulins or steroids infusion [7]. Based on a combination of clinical, imaging, and laboratory findings, MOG antibody testing should be performed in patients with a high risk of MOGAD and/or in the case of findings that are atypical for MS and NMSOD. Particularly, the analysis should be considered in patients with an NMOSD phenotype but with negative AQP4-IgG. Authors of the MOG-IgG testing proposals stated that these tests are primarily intended for the use in adults and adolescents. New MRI pictures of cortical lesions and immunopathological findings have been reported after recommendations had been published in 2018, so the original summary was slightly modified (Table 2) [7,36,65].

## 8. MOGAD Treatment

MOGAD is a relatively new condition with low prevalence, age-related differences in the presentation and a broad spectrum of clinical symptoms, hence conducting controlled multicenter, large treatment trials might be complicated and in fact, such study has not been performed to date. 

There are no evidence-based guidelines for the acute and maintenance immunotherapy of MOGAD. Since this entity has been separated from MS and NMO spectrum, it is not surprising that similar treatment approaches have been attempted. The drug of choice for the acute exacerbations management is high-dose intravenous methylprednisolone (IVMP, usually 0.5–2.0 g for 5–10 days). Patients with more severe (or steroid unresponsive) attacks were treated early with plasma exchange and intravenous immunoglobulin (IVIG). Studies conducted on general populations with MOGAD have shown high effectiveness of these therapies. More than 50% of subjects with ON treated with IVMP reached almost full recovery. There are also occasional reports about complete recovery in patients with brainstem and cortical encephalitis [70,71]. Unfortunately, the risk of worsening increased after steroid discontinuation or dose reduction [45,49,72,73]. The number of reported second relapses was substantial. In the UK trial the next attack occurred in about 50% of cases over 2 years [45]. In another frequently cited study, relapses were noticed in as many as 80% of patients during a follow-up [49]. On the contrary to MS, disability progression in MOGAD is relapse-depended, so in a number of patients maintenance therapy should be initiated, especially in those with positive anti-MOG antibodies. Studies proved that higher antibody levels and persistent seropositivity over time were associated with a higher risk of a relapsing disease course and increased disability [48]. Therapeutic strategies to prevent MOGAD attacks, which have been explored so far, involved mainly standard immunomodulating and immunosuppressive therapies, such as oral corticosteroids (OCS), rituximab (RTX), azathioprine (AZA), methotrexate (MTX), mycophenolate mofetil (MMF), and repeated cycles of IVIG. The effect of classical MS disease modifying drugs (DMD) (interferon beta, glatiramer acetate, natalizumab) was also analyzed, as discussed below.

A prolonged gradual taper off OCS, with their anti-inflammatory action (reduction of overall antibody production) is a common and convenient approach for relapse prevention. In the UK trial, patients with treatment longer than three months (oral prednisolone 10 mg daily) had about 25% risk of a relapse, whereas in patients without or with shorter (up to 3 months) immunosuppression, this risk was almost doubled and stood at 47% [4,52]. The effectiveness of low dose OCS (oral prednisolone 10 mg daily) was confirmed by other groups [49,71,72,73]. However, due to the possible devastating side effects of long-term steroid administration, a switch to a steroid-sparing medication might be considered. Patients treated with immunosuppressants such as AZT (adjunctively with oral steroids), MMF and MTX presented better outcome in terms of relapses and disability [52,72,73,74,75]. The efficacy of RTX is not constant. Similarly to AQP4-positive NMO patients, new relapses within the few weeks following the first infusion were observed in about 30% of MOGAD patients, despite an effective biological effect of this agent [76]. 

IVIG infusions gave promising results regarding effectiveness. Initial studies and case reports showed a good therapeutic response of MOGAD subjects [73,74,77]. Very recently, a relatively large multicenter (but still retrospective) study on seventy MOGAD patients compared maintenance therapy with RTX, AZA, MMF and IVIG. The lowest annualized relapse rate was observed in the IVIG-treated group. The proportion of patients worsening was 20% in cases treated with IVIG, 59% with AZA, 61% with RTX and 74% with MMF [78].

Glatiramer acetate and natalizumab have both been shown to be ineffective, also interferon beta injections were not successful, moreover, it increased the disease activity [52,74].

## 9. Conclusions

The discovery of novel antibodies remarkably refined our thinking about inflammatory demyelinating diseases of the CNS. Nowadays, it is rather obvious that they might have a diverse range of immunological processes. The research of MOGAD, which has rapidly advanced in recent years, is an excellent proof of such statement. Experimental and clinical studies have resolved a number of concerns and substantially altered the concept of the disease. Previously considered as a variant of NMOSD, characterized by frequent involvement of the optic nerves and spinal cord, MOGAD is now recognized as an autonomous, antibody-mediated inflammatory demyelinating disorder, most frequently with relapsing course and a variety of clinical manifestations. As it is a rare condition, researchers should still focus on the different aspects of the disease: its pathophysiological mechanisms, the identification of markers of the disease status and outcome, the incidence in various regions and patients populations. All of the above should form the basis for development and elaboration of the MOGAD diagnosis criteria to be ultimately approved by experts. Finally, randomised controlled trials should be conducted to establish the most optimal way of care and treatment strategy.

## Figures and Tables

**Table 1:** Cerebrospinal fluid analysis in patients with MOGAD, NMOSD, and MS [56,57,58,59].

	NMO	MS	MOGAD
Oligoclonal bands (OCB)	Typically absent (present in approx. 15–30% of NMO patients, they can occur transiently)	Positive in approx. 85–90% of MS patients (they do not disappear or change in the course of the disease or following treatment)	CSF-restricted OCB unusual, positive in a minority of samples (13.2%)
IgG index	Usually elevated	Elevated > 0.7 (typically > 1.7) in approx. 70% of MS patients (decreases following steroid treatment)	elevated in a minority of samples (8%)
Total protein	Elevated (100–500 mg/dL) in approx. 25–30% of NMO patients	Within normal limits or > 40 mg/dL in approx. 15% of MS patients	elevated in approx. 44% of samples (range 45.3–176 mg/dL)
Cytosis	>50/mm^3^ (at the time of the attack in approx. 30–80% of NMO patients)	>5/mm^3^ (rarely above 50/mm^3^) in approx. 30% of patients	Pleocytosis present at least once in> 57% of samples, > 50 cells/mm^3^ in about 19% of cases
Cell type	Neutrophil-predominant pleocytosis, with the presence of eosinophils	Mononuclear cells; lymphocyte-predominant	Lymphocytes and monocytes, neutrophils present in at least 43% of cases; eosinophils and basophils–very rare

**Table 2:** MOG -IgG testing in MOGAD diagnosis.

Clinical	
**Optic neuritis (ON)**	- bilateral acute ON - high ON frequency or disease mainly characterized by recurrent ON, CRION - severe visual deficit/blindness in acute ON
Myelitis	- severe or frequent episodes of acute myelitis - continuous sphincter and/or erectile disfunction
ADEM brainstem/cortical encephalitis	- acute respiratory insufficiency - disturbance of consciousness - abnormal behavioral changes - epileptic seizures - unexplained intractable nausea and vomiting or intractable hiccup - (area postrema syndrome)
Fundoscopy	- prominent papilledema/papillitis/optic disc swelling during acute ON
Others	- the onset of disease from 4 days to 4 weeks after vaccination - co-existing teratoma or NMDAR encephalitis (low evidence)
**Laboratory**	
MRI image	- longitudinally extensive optic nerve lesion (>1/2 of the length of the pre-chiasmal optic nerve, T2 or T1/Gd) - peri-optic Gd enhancement during acute ON spinal cord - longitudinally extensive spinal cord inflammatory lesion (≥3 VS, adjacent) LETM - longitudinally extensive spinal cord atrophy (≥3 VS, adjacent) in patients with a history of acute myelitis - conus medullaris lesions, especially if present at the onset - a normal supratentorial image in patients with acute ON, myelitis and/or brainstem encephalitis - abnormal brain image but no lesion adjacent to a lateral ventricle that is ovoid/round, no Dawson’s finger-type or juxtacortical U fiber lesions - large, confluent T2 brain lesions typical for ADEM - unilateral cortical T2-FLAIR hyperintensity without the involvement of the adjacent juxta-cortical white matter (FLAMES)
CSF	- neutrophilic CSF pleocytosis or CSF WCC > 50/μL - no CSF-restricted OCB at first or any follow-up examination
Histopathology	- primary demyelination with intralesional complement and IgG deposits (“pattern II of MS”) - the perivenous disseminated inflammatory demyelination (with MOG-dominant myelin loss, preserved oligodendrocytes, CD4- dominant T cell infiltration, perivascular MOG-laden macrophages)
Treatment response	- re-occurrence of symptoms after tapering of steroids - increase in relapse rate following treatment with IFN-beta or natalizumab in patients diagnosed with MS (low evidence)

Abbreviations: ADEM acute disseminated encephalomyelitis, CD4 cluster of differentiation 4, CRION chronic relapsing inflammatory optic neuropathy, CSF cerebrospinal fluid, FLAMES (FLAIR-hyperintense lesions in anti-MOG-associated encephalitis with seizures), Gd gadolinium, IgG immunoglobulin G, IFN-beta interferon beta, LETM longitudinally extensive transverse myelitis, MOG myelin oligodendrocyte glycoprotein, MRI magnetic resonance imaging, MS multiple sclerosis, NMDA-R N-methyl-D-aspartate receptor, OCB oligoclonal IgG bands, ON optic neuritis, VS vertebral segments, WCC white cell count. Modified and adapted from Jarius et al., (open access under the terms of the Creative Commons Attribution 4.0 International License (http://creativecommons.org/licenses/by/4.0/) [7,69].

## Data Availability

Data sharing not applicable.
